# Energy Requirement for Elderly CKD Patients

**DOI:** 10.3390/nu13103396

**Published:** 2021-09-27

**Authors:** Claudia D’Alessandro, Domenico Giannese, Monica Avino, Adamasco Cupisti

**Affiliations:** 1 Department of Clinical and Experimental Medicine, University of Pisa, 56125 Pisa, Italy; dalessandro.claudia@gmail.com (C.D.); domenico.giannese@phd.unipi.it (D.G.); 2 Postgraduate School of Nephrology, Vita Salute San Raffaele University, 20121 Milan, Italy; avino.monica@hsr.it

**Keywords:** energy expenditure, indirect calorimetry, CKD, dietary energy intake, diet

## Abstract

The correct management of energy intake is crucial in CKD (chronic kidney disease) patients to limit the risk of protein energy wasting especially during low-protein regimes, but also to prevent overweight/obesity. The aim of this study was to assess the energy requirement of older CKD patients using objective measurements. This cross-sectional study enrolled 67 patients (aged 60–86 years) with CKD stages 3–5 not on dialysis, all of whom were metabolically and nutritionally stable. All patients underwent indirect calorimetry and measurement of daily physical activity level expressed by the average daily Metabolic Equivalent Task, using an accelerometer, in order to measure total energy expenditure (mTEE). Estimated TEE (eTEE) was derived from predictive equations for resting energy expenditure and physical activity levels coefficients. The mTEE were lower than eTEE-based on Harris–Benedict or Schofield or Mifflin equations (1689 ± 523 vs. 2320 ± 434 or 2357 ± 410 or 2237 ± 375 Kcal, *p* < 0.001, respectively). On average mTEE was 36.5% lower than eTEE. When eTEE was recalculated using ideal body weight the gap between mTEE and eTEE was reduced to 26.3%. A high prevalence of a sedentary lifestyle and reduced physical capabilities were also detected. In conclusion, our data support the energy intake of 25–35 Kcal/Kg/d recently proposed by the NKF-KDOQI (National Kidney Foundation-Kidney Disease Improving Quality Initiative) guidelines on nutritional treatment of CKD, which seem to be more adequate and applicable than that of previous guidelines (30–35 Kcal/Kg/d) in elderly stable CKD patients with a sedentary lifestyle. According to our findings we believe that an energy intake even lower than 25 Kcal/Kg/d may be adequate in metabolically stable, elderly CKD patients with a sedentary lifestyle.

## 1. Introduction

The prevalence of protein energy wasting (PEW) exceeds 20–25% in people with mild to moderate chronic kidney disease (CKD) and increases to 30–40% in severe CKD. PEW is associated with a pro-inflammatory status and an increase in protein catabolism, loss of muscle mass, poor appetite, scarce physical performance and increase mortality risk [1,2].

On the other hand, epidemiology of CKD has changed in the last decades: the majority of CKD patients is overweight or obese, with high prevalence of diabetes and other comorbidities. All these conditions represent independent risk factors for progression of CKD and cardiovascular damage [3,4].

Proper management of energy prescription is crucial in CKD patients to prevent the onset of PEW, especially for patients on low-protein regimes but also to prevent the latest trend of overweight/obesity in the CKD population. The high prevalence of sedentary lifestyle that characterizes elderly CKD patients greatly affects and complicates energy requirement calculation.

Energy intake of 30–35 Kcal/Kg ideal body weight (IBW) per day, has been recommended by guidelines for many years. It is noteworthy that these values came from outdated nitrogen balance studies performed in the 1980′s [5] on patients who were very different from the current ones. In the real-life clinical practice, such high energy intakes have been rarely detected. In fact, the assessments of energy intake derived from dietary recalls analysis were generally lower than recommended, but the prevalence of malnutrition was very rare with respect to the theoretical energy deficit, even for those on low protein regimes [6]. The prevalent interpretation of this discrepancy was the under-estimation of dietary recalls rather than the over-estimation of the energy requirements. According to these observations, the very recent NKF-KDOQI guidelines on nutrition lower the recommended range of energy intake to 25–35 Kcal/Kg/d [7] for CKD patients in stages 3–5 not on dialysis.

Although the overall prevalence of CKD is roughly 10% in the general population, it increases with age, affecting more than one third of all individuals older than 65 years; the age of patients attending CKD clinics is 73 years [8,9,10,11]. In this population, CKD is associated with several comorbid conditions, a higher risk of cardiovascular disease and increased prevalence of frailty, disability and malnutrition [12].

Energy management in older adults is often complicated by many factors that affect their dietary habits: chewing problems, lack of appetite, depression, low income and scarce physical capacity [13]. Another cause of concern is the high prevalence of sedentary lifestyle that characterizes elderly CKD patients and lowers the energy requirement [14,15].

Therefore, the correct management of energy prescription is important to avoid insufficient amount of calories predisposing to PEW and even negative nitrogen balance, as well as to avoid excess energy intake, leading to overweight or obesity. In the daily clinical practice, energy expenditure is usually estimated by predictive equations and subjective evaluation of physical activity. However, it is preferable to use objective measurements in the single patient whenever possible. Currently, total energy requirement can be assessed by determination of resting energy expenditure by indirect calorimetry plus objective evaluation of physical activity by means of accelerometry.

The aim of this study was to determine the total energy requirement of older CKD patients by objective methods, namely indirect calorimetry plus accelerometry, compared to its estimation by predictive equations. In addition, we evaluated the discrepancy between actual dietary energy intake and measured or estimated total energy requirement.

## 2. Subjects and Methods

Sixty-seven out-patients (11 f, 56 m) aged 60–86 years affected by CKD stage 3–5ND, metabolically and nutritionally stable, entered the study during the period of November 2017–October 2019.

Patients with severe cardiac failure (Stage IV NYHA), respiratory insufficiency, cancer, dementia, psychiatric or neurologic diseases, inflammatory systemic diseases or patients with a Barthel Index and/or Karnofsky score < 100 or those who did not give their consent to the study were excluded.

All recruited patients underwent indirect calorimetry and evaluation of daily physical activity by accelerometry. In addition, all patients received an equation-based estimation of their energy expenditure. A nutritional and functional assessment was also performed to assess body composition and performance capacity: it included anthropometry, bioimpedance analysis, biochemistry and functional tests. Dietary habits were also assessed by three-day dietary record combined with a dietary recall.

### 2.1. Resting Energy Expenditure

Resting energy expenditure (REE) was measured by indirect calorimetry with a hand-held desktop calorimeter (Fitmate GS, Cosmed, Rome, Italy) using the dilution technique. The Fitmate accuracy was validated against the gold standard Douglas Bag. The oxygen sensors were automatically calibrated before each measurement. Patients arrived at clinic at 8.30 a.m. after 12 h of fasting. They were previously instructed not to exercise the day before the test. After their arrival they were asked to rest for at least 20 min laying on a bed. Then, a ventilated hood (canopy) was placed over their head and shoulders making sure that air did not enter from the outside or leak from the system. The canopy allowed patients to breathe freely. The test lasted 20 min with measurements of oxygen consumption at 1 min intervals. REE was calculated from O_2_ consumption using a modified Weir equation [16,17].

The predictive equations used for the estimation of REE were: the Harris–Benedict equation [18], Schofield equation [19] and Mifflin–St Jeor equation [20]; a novel equation proposed by Xu et al. [21] for estimating resting energy expenditure in CKD patients was also applied. Physical activity level (PAL) coefficients were attributed according to the FAO/WHO/UNU expert committee (1985) [22]

### 2.2. Measurement of Physical Activity

A Sense-wear Armband (SWA, BodyMedia, Pittsburgh, PA, USA) was applied as an objective measurement of spontaneous physical activity in order to calculate total energy expenditure (TEE).

Patients were advised to wear the SWA on the upper non-dominant arm, in the middle tract between the acromion and olecranon processes, over the triceps muscle. They were instructed to wear the SWA for three consecutive days, while continuing their usual daily life, being careful to remove it only during shower or water activities. The SWA collects physiologic data through multiple sensors, in particular a two-axis accelerometer, a heat flux sensor, a skin temperature sensor, a near-body ambient temperature sensor and a galvanic skin response sensor; data were processed using a specific software (InnerView™ Research Software, Version 6.1).

The integrated detections from the biaxial accelerometer and the heat-related sensors have shown to provide additional information that cannot be obtained from movement sensors alone; they have high sensitivity in detecting even little changes in energy expenditure associated with complex activities.

The SWA has been validated both under laboratory conditions and under free living conditions, and it has potential advantages in accuracy when compared with traditional accelerometry-based devices [23,24,25,26].

The SWA allows the assessment of physical activity as measured by the Metabolic Equivalent Task (MET) [27]. The MET is a way of quantifying the intensity of physical activity and the associated energy expenditure and it is defined as the ratio of metabolic rate during a specific physical activity to the reference metabolic rate at rest, set by convention at 3.5 mL O_2_ × Kg^−1^ × min^−1^. The metabolic equivalent can be converted into Kilocalories using the following equation: MET = Kcal/h/Kg b.w; 1 MET is the energy expended at rest. Therefore, two METs indicates that the energy expended is twice than that at rest [28,29].

The measurements were calculated on a daily basis and included: average daily METs, number of steps, the minutes spent in physical activity with intensity > 3 MET/min, between 6 and 9 MET/min, or >9 MET/min.

The average daily METs (adMETs) represents an objective measure of physical activity and allows quantification of the energy expenditure during physical activity with respect to resting energy expenditure. Hence, adMETs values may be considered as a measure of the average daily activity level; in older adults an adMETs value < 1.5 METs defines a sedentary condition [30].

### 2.3. Measurement of Performance Capacity

All the patients performed the 30” Sit-to-Stand Chair Test (30” STS) and the Six-Minute Walking Test (6MWT). The 30” STS is a validated test which allows us to assess lower-extremity strength in adults [31,32]. The patient seated in a chair with the arms crossed on the chest and hands resting against their shoulders. The score corresponds to the number of times the person can stand up from a sitting position during the time period of 30” without the help of their arms. [33].

The 6MWT was performed according to the American Thoracic Society’s guidelines [34]. The test measures the distance a subject is able to walk over six minutes on a hard, flat surface. After resting for 10 min, the subject walks along a path with marked turning points. The patient is allowed to self-pace and rest as needed [34].

### 2.4. Assessment of TEE

Measured TEE (mTEE) was determined by indirect calorimetry multiplied by adMETs values as assessed by SWA. Estimated TEE (eTEE) was obtained by predictive formulas multiplied by PAL coefficients.

### 2.5. Anthropometry

Body weight was assessed on a mechanical scale with the patient wearing light clothes and no shoes. Height was measured with a stadiometer. Body mass index (BMI) was calculated as body weight (Kg)/height^2^ (m^2^). Ideal body weight was defined with the reverse equation for BMI (IBW = ideal BMI × height^2^) according to the recommendation of the UK Renal Association (Nutrition in CKD) that is Ideal BMI = 20 if current BMI was <20 Kg/m^2^; Ideal BMI = 25 if current BMI was >25 Kg/m^2^; in those patients with BMI between 20 and 25 Kg/m^2^, calculations were performed using the current body weight [35]. Other measures included waist and hip circumferences.

Body composition analysis was estimated using a Bioelectrical Impedance single frequency Analyzer (BIA/STA, Akern, Florence, Italy) with a distal, tetrapolar technique, delivering an excitation current at 50 kHz.

Impedance (Z) represents the force that interferes with the flow of electric current and is given by the vectorial sum of the resistance (Rz) and the reactance (Xc), the two bioelectric parameters given by the body analyzer.

The phase angle is the most immediate bioelectric index resulting from a proportion between resistance and reactance according to this formula: phase angle = Arctang (Xc/Rz) × 180 × π. It reflects hydration status and soft tissue cellular mass. Reduced phase angle value reflects increased extra- to intra-cellular water ratio as well as reduced body cell mass. Body cell mass (BCM) is derived from bio-impedance analysis and body cell mass index (BCMI) is consequently calculated. The phase angle is considered a strong predictor of survival in CKD in patients on conservative therapy, on peritoneal dialysis or on maintenance hemodialysis, in HIV or cancer patients and other chronic diseases [36,37,38,39].

### 2.6. Biochemistry

Biochemistry included serum levels of creatinine, blood urea nitrogen (BUN), phosphorus, calcium, albumin, bicarbonate, parathyroid hormone (PTH) and hemoglobin. Tests were performed using standard laboratory methods. Glomerular filtration rate (eGFR) was estimated using the CKD-EPI formula [40]. Urinary sodium, phosphate, and urea were measured on 24 h urine samples. Protein catabolic rate was calculated by the Maroni–Mitch formula in order to estimate dietary protein intake [41].

### 2.7. Assessment of Dietary Energy Intake

The current daily energy intake was assessed using a 3-day record combined with a dietary recall, that is the patient first collects his/her dietary habits in a dietary record then, upon delivery, the renal dietician checks the dietary record and interviews the patient to clarify any uncertainties or incomplete records.

The actual amount of food consumed was identified by weighting or through the use of photographic images of portions (as actual size) included in an atlas photo. Composition data were obtained using latest food composition tables of the European Institute of Oncology [42].

### 2.8. Statistical Analysis

Results are reported as the mean ± standard deviation, or median and interquartile range, when applicable. Categorical data were described by absolute and relative frequency. Normality of the distribution data was assessed by a Shapiro–Wilk test. Statistical analysis was performed using a t-test for un-paired samples (two-tailed) or one-way ANOVA. To compare quantitative variables with continuous data Pearson’s correlation analysis was performed. Bland–Altman plot was used to describe the agreement between mTEE and eTEE. Differences were considered as statistically significant when *p* < 0.05. All data processing was carried out by SPSS v.26 technology.

## 3. Results

Table 1 resumes the clinical features, anthropometry and physical function of the studied patients. According to the selection criteria, all the patients were metabolically and nutritionally stable, and had normal physical function. The 23.5% of studied patients were type 2 diabetics.

The REE as assessed by indirect calorimetry (1315 ± 233 Kcal/d) was significantly lower than that calculated by Harris–Benedict or Schofield or Mifflin equations (1545 ± 234 or 1568 ± 205 or 1492 ± 208 Kcal/d, respectively, *p* < 0.001), as well as by the Xu equation (1453 ± 196 Kcal/d).

Physical activity levels as assessed by adMETs were lower than estimated PAL coefficients (1.28 ± 0.23 vs. 1.49 ± 0.10, respectively, *p* < 0.001).

**Figure 1:**
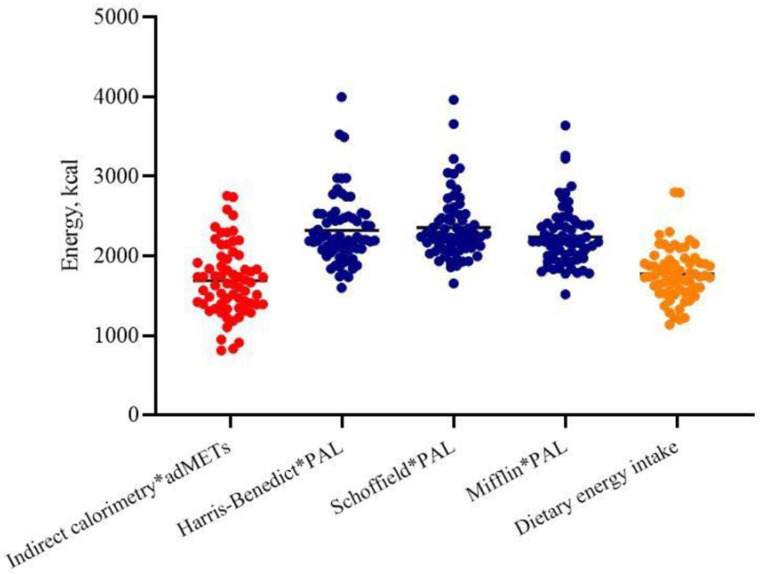
Total energy expenditure measured by indirect calorimetry *adMETs (red points) and estimated by predictive equations *PAL (blue points). The ANOVA (*p* = 0.0001). The yellow points represents dietary energy intake derived from dietary recalls.

Hence, the TEE calculated using indirect calorimetry multiplied by SWA-derived adMETs was lower than that estimated by Harris–Benedict or Schofield or Mifflin equations multiplied by standard PAL coefficients (1689 ± 523 vs. 2320 ± 434 or 2357 ± 410 or 2237 ± 375 Kcal, *p* < 0.001, respectively) (Figure 1); also, when calculated using the Xu formula, TEE (2180 ± 367 Kcal) was similar to the eTEEs obtained by the other formulas.

**Figure 2:**
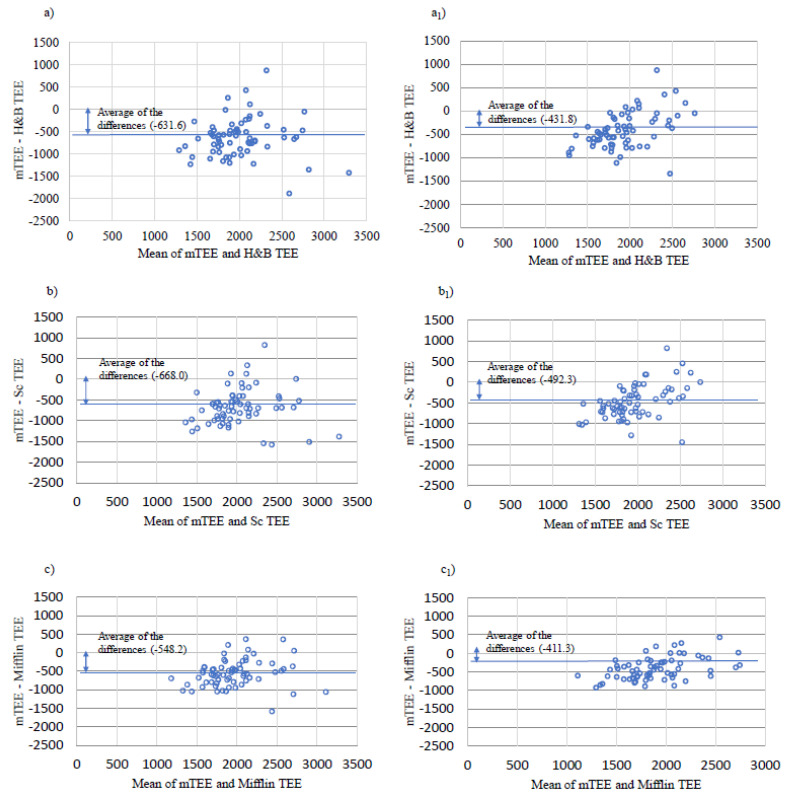
Bland–Altman plots. mTEE: total energy expenditure measured by Indirect calorimetry *adMETs as assessed by the Sensewear Armband) H&B TEE: total energy expenditure estimated by the Harris–Benedict equation *PAL; Sc TEE: total energy expenditure estimated by the Schoffield equation *PAL; Mifflin TEE: total energy expenditure estimated by the Mifflin equation *PAL using actual body weight (**a**–**c**) and using ideal body weight (**a_1_**–**c_1_**) in the predictive equations.

Figure 2 shows the Bland–Altman graphs plot that represents the differences between mTEE and eTEE. On average eTEE using the Harris–Benedict, Schoffield and Mifflin equations and PAL coefficients overestimated mTEE by 632 ± 441, 668 ± 435 and 548 ± 363 Kcal, respectively, (Figure 2a–c).

When eTEE was recalculated using ideal body weight instead of current body weight in the predictive equations, the gap between mTEE and eTEE was significantly reduced to 432 ± 358 Kcal *p* = 0.0058, 492 ± 374 Kcal *p* = 0.021 and 411 ± 303 Kcal *p* = 0.0001, respectively (Figure 2(a_1_–c_1_)).

**Figure 3:**
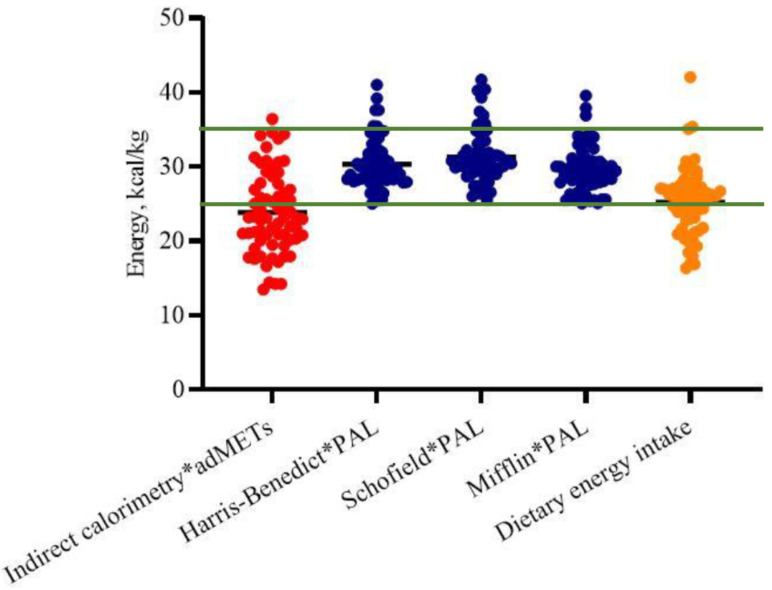
Measured and estimated total energy expenditure, and dietary energy intake reported per kilogram of ideal body weight. Green lines correspond to NKF-KDOQI 2020 recommendations [7].

Figure 3 shows TEE, measured and estimated, normalized by ideal body weight. Measured TEE was lower than that recommended by the recent NKF-KDOQI guidelines (green lines) in more than one half of the patients. According to the selection criteria, all the patients were metabolically stable and free from neurological or muscular diseases, and from physical function impairments, but spontaneous physical activity resulted as scarce both in terms of daily distance walked and intensity (Table 1) denoting high prevalence of a sedentary lifestyle. On the basis of the average number of daily steps, 36.7% of patients were classified as “sedentary”, 32.3% as “low active” and 19.1% as “somewhat active” [43].

Accordingly, physical capacities as assessed by the 6MWT and sit-to stand test also resulted quite low (Table 1). In the 6MWT, the 46% of patients walked a distance shorter than expected for age and sex, and the performance at the STS30 test was lower than expected in the 83% of patients.

Measured REE positively correlated with adMETs (r = 0.471, *p* < 0.001) and with the performance at the sit-to-stand (r = 0.458, *p* < 0.001). adMETs was negatively associated with the percentage of fat mass (r = −0.55, *p* < 0.001) while it was positively associated with body cell mass (r = −0.39, *p* < 0.002), phase angle (r = 0.46, *p* < 0.001) and the percentage of free fat mass (r = 0.55, *p* < 0.001).

## 4. Discussion

In the present study, the TEE measured by indirect calorimetry and multiplied by adMETs values as assessed by a physical activity detector device is significantly lower than that estimated by predictive equations and multiplied by PAL coefficients. The mTEE provided lower values than the energy intake recommended for CKD patients. However, both the mTEE and estimated energy intake are similar to those reported in previous studies on CKD and ESRD populations [44,45]. Using the ideal body weight instead of the current body weight makes the results of the predictive formulas closer to that of indirect calorimetry.

In most studies, predictive equations were used to estimate REE, but only a few researchers have questioned whether they may also be appropriate for patients with CKD. Kamimura et al. in 2011 compared REE from Harris–Benedict and Schofield equations with indirect calorimetry in 81 healthy subjects and 281 renal patients (124 on conservative treatment, 99 on hemodialysis and 58 on peritoneal dialysis) and observed that predictive equations used for healthy individuals overestimated the REE of CKD patients, with a discrepancy that decreased in the presence of comorbidities [46]. In the literature the question has been investigated as to whether the various predictive equations that have been formulated to estimate the REE can be applied to patients with chronic diseases [47,48].

Recently Xu et al. have developed and validated a REE predictive equation for CKD patients based on REE measured using indirect calorimetry [21]. The equation still differed from measured REE but it approached the latter significantly better than the Harris–Benedict, WHO and Schofield equations. The formula takes into account sex, age, weight and presence/absence of diabetes. When the Xu formula was applied to our population to estimate TEE, we found an overestimate compared to measured TEE. The overestimation was lower than that from the most used predictive equations.

The kidneys have important metabolic functions and perform oxygen-dependent activities: in healthy individuals, the energy expenditure of the kidneys is approximately 7% of the TEE at rest [49]. Kidney failure is associated with reduction in cellular metabolism, so it is possible that patients with advanced CKD have a reduced REE [50]. More interestingly, the CKD population has changed over time: patients are older and affected by a number of comorbidities. Additionally, overweight or obesity are highly prevalent. As a whole, the tendency for a sedentary lifestyle is a very typical finding in the CKD population and this invariably lowers TEE and energy requirements. Therefore, the questions arise as to whether recommendations of high energy intake (>30–35 Kcal/Kg/d) are still appropriate for older people with CKD that are overweight or obese.

The progressive loss of kidney function is associated with a number of disorders such as metabolic acidosis, insulin resistance, inflammation, increased protein catabolism, secondary hyperparathyroidism, but this does not seem to increase the REE [51]. Some studies compared the REE of CKD patients with that of healthy subjects, but they reported contradictory results: some of them found a decrease in REE [50,52] while others did not find any difference with healthy subjects [44,49]. Avesani et al. [52], compared REE by indirect calorimetry in 45 renal patients with 45 healthy subjects, 20 males and 25 females in each group. The REE in non dialysis-dependent CKD patients was significantly lower than in the control group, especially in females. The authors hypothesized that this could be linked both to a decrease in food intake and to various metabolic disorders due to the decreased kidney function.

REE was positively associated with the average physical activity level (adMETs) which was expected as it is known that physical activity level significantly affects the basal metabolic rate, namely a sedentary subject usually has a lower basal metabolism than a subject who practices constant physical activity [53]. As a whole, our data suggested thatm for elderly and sedentary CKD patients, the energy requirement may be lower than previously recommended. These findings support the recent guidelines on renal nutrition which have reduced the lower limit of dietary energy intake from 30 to 25 Kcal/Kg/d. It can be speculated that preventing extra energy intake may counteract overweight or obesity, which in turn could cause additional functional impairment and increase morbidity and mortality risk. It is noteworthy that current dietary energy intake, as assessed by dietary recalls, is quite similar to the mTEE and much lower than the eTEE calculated from predictive equations. 

In order to limit the bias of underestimation, we prefer to ask the patients to register a dietary record, providing all the necessary information for the correct compilation. Patients completed it at home and, when completed, the dietary record was handed back to the dietician who interviewed the patient to verify the adequacy of the reporting.

In several reports present in the literature the energy intakes calculated by dietary recalls were generally lower than the recommended levels, but the prevalence of PEW signs was quite rare: it occurred also in the MDRD trial [6]. The most accepted interpretation was an under-estimation of dietary recalls rather than an over-estimation of the energy requirement. Moreover, this is in keeping with data from the literature where effective dietary intakes were lower than recommended in well-nourished patients as well [7].

The strength of the study consists in coupling the measurement of REE by indirect calorimetry with the measurement of physical activity by accelerometer: it allowed us to obtain an objective measure of TEE. Unfortunately, limitations exist, such as the cross-sectional, single center design of the study, including a quite low number of patients. These aspects prevent us from drawing solid conclusions and detract from transferability of the results. Future research addressing the measurement of TEE in CKD patients should be planned in order to be able to prescribe the adequate energy intake in older and younger CKD patients.

In conclusion, our data support the energy intake of 25–35 Kcal/Kg/d recently proposed by the recent NKF-KDOQI guidelines on nutritional treatment on CKD, which seems to be more adequate and applicable than that of previous recommendations (30–35 Kcal/Kg/d). According to our findings we believe that an energy intake even lower than 25 Kcal/Kg/d may be adequate in a number of elderly stable CKD patients with a sedentary lifestyle.

## Figures and Tables

**Table 1:** Clinical features, anthropometry and physical function of the studied patients.

Parameter	Mean ± SD
Age, years	67.5 ± 12.7
Weight, kg	80.0 ± 13.0
BMI, kg/m^2^	28.2 ± 4.0
Waist circumference, cm	104 ± 14.1
sUrea, mg/dL	81.1 ± 30.1
sCreatinine, mg/dL	2.5 ± 1.2
eGFR, ml/min × 1.73 m^2^	32.0 ± 13.8
sSodium, mEq/L	141 ± 2.5
sPotassium, mEq/L	4.7 ± 0.6
sCalcium, mg/dL	9.4 ± 0.5
sPhosphate, mg/dL	3.3 ± 0.5
sBicarbonate, mEq/L	24.5 ± 3.0
sTotal Protein, g/dL	7.2 ± 0.5
sAlbumin, g/dL	4.2 ± 0.4
Hemoglobin, g/dL	13.3 ± 1.6
Hematocrit, %	39.5 ± 1.5
Phase angle, °	5.1 ± 1.2
BCMI, kg/m^2^	9.4 ± 1.7
Steps, no./d	6476 ± 3800
Walked distance, m/d	5213 ± 3586
Average daily METs	1.28 ± 0.23
Time spent in PA > 3 METs, min/d	61 ± 58
Time spent in PA > 6 METs, min/d	2.9 ± 7.5
Time spent in PA > 9 METs, min/d	0.1 ± 0.1
Time spent lying down, min/d	527 ± 113
6-Minute walking test, m	337 ± 101
Sit-to-stand 30”, no. of stands	11.2 ± 2.9

BCMI: body cell mass index. PA: physical activity. MET: metabolic equivalent task.
